# A Comprehensive Survey on Spectrum Sensing in Cognitive Radio Networks: Recent Advances, New Challenges, and Future Research Directions

**DOI:** 10.3390/s19010126

**Published:** 2019-01-02

**Authors:** Youness Arjoune, Naima Kaabouch

**Affiliations:** School of Electrical Engineering and Computer Science (SEECS), University of North Dakota, Grand Forks, ND 58202, USA; naima.kaabouch@ndus.edu

**Keywords:** cognitive radio, spectrum sensing, narrowband sensing, wideband sensing, compressive sensing, machine learning

## Abstract

Cognitive radio technology has the potential to address the shortage of available radio spectrum by enabling dynamic spectrum access. Since its introduction, researchers have been working on enabling this innovative technology in managing the radio spectrum. As a result, this research field has been progressing at a rapid pace and significant advances have been made. To help researchers stay abreast of these advances, surveys and tutorial papers are strongly needed. Therefore, in this paper, we aimed to provide an in-depth survey on the most recent advances in spectrum sensing, covering its development from its inception to its current state and beyond. In addition, we highlight the efficiency and limitations of both narrowband and wideband spectrum sensing techniques as well as the challenges involved in their implementation. TV white spaces are also discussed in this paper as the first real application of cognitive radio. Last but by no means least, we discuss future research directions. This survey paper was designed in a way to help new researchers in the field to become familiar with the concepts of spectrum sensing, compressive sensing, and machine learning, all of which are the enabling technologies of the future networks, yet to help researchers further improve the efficiently of spectrum sensing.

## 1. Introduction

The dramatic growth in the number of wireless devices alongside the static management of the radio spectrum have created a shortage of available radio spectrum [1]. Over 50 billion wireless devices will be connected by 2020, all of which are likely going to demand access to the Internet [2]. The static management of the radio spectrum is no longer efficient enough to grant access to all these devices. With this allocation, some portions of the radio spectrum are heavily used while some others are not or rarely used. Not sharing the radio spectrum among users can result in the creation of unwanted denial of service events. The scarcity of the radio spectrum is thus one of the most urgent issues at the forefront of future network research that has yet to be addressed.

One solution to these and other challenges is to use cognitive radio technology, which has undergone extensive investigation by the research community for almost two decades [3]. Cognitive radio technology allows wireless devices to sense the radio spectrum, decide about the state of the frequency channels, and reconfigure their communication parameters to meet quality-of-service requirements while minimizing their energy consumption [4]. These devices can use unlicensed bands as well as licensed bands when their licensed primary users are not active, preventing adverse interference.

Over the last decade, a number of sensing techniques have been proposed which can be classified into two categories: narrowband and wideband. Narrowband sensing analyzes one frequency channel at a time while wideband sensing analyzes a number of frequencies at a time. Examples of the former include energy detection [5,6,7,8,9,10,11,12,13,14,15,16,17,18,19,20], cyclostationary features detection [21,22,23,24,25,26,27], matched filter detection [28,29,30,31], covariance based-detection [32,33,34,35,36,37,38,39], and machine learning-based sensing [40,41,42,43,44,45,46,47,48,49,50,51]. In the latter, the spectrum is usually divided into multiple sub-bands and then they are sensed, either sequentially or simultaneously, using the narrowband sensing techniques. Sequential-sensing approaches are ineffective because they require longer times and higher energy due to the use of high-rate analog-to-digital converters (ADC), which is both costly and impractical for timely communications. Simultaneous sensing schemes require a large number of sensors and joint synchronized function, increasing the complexity of a given implementation [52].

A way forward is to decrease the high number of samples acquired using compressive sensing [53,54,55,56]. A number of spectrum sensing projects have shown that most frequency channels of the wideband spectrum are used scarcely or not at all [57,58,59,60]. Hence, the wideband spectrum signals can be regarded as sparse signals, a characteristic that has motivated researchers to investigate the use of sub-Nyquist, or compressive sensing, to speed up the process of the wideband spectrum sensing [50,51,52,53,54,61,62,63,64,65,66]. 

Compressive sensing recovers the original sparse signal from only a few measurements. It involves three main processes: sparse representation, coding with the sensing matrix/measurement, and decoding, also called sparse recovery [53,54,55,56]. To apply compressive sensing, signals are required to be sparse in a given domain and the sensing matrix has to satisfy the restrict isometry property (RIP), or it must have a small mutual incoherence to guarantee the exact recovery of the original sparse signal. The optimal number of measurements depends on the sparsity level of the wideband signal, the measurement matrix, and the recovery techniques being used [54,56,67]. Some authors have investigated how to estimate the sparsity level of the wideband signal and then adapt the number of required measurements. Other authors have proposed blind compressive sensing techniques for wideband spectrum sensing, which do not require any prior knowledge of the sparsity level of the wideband signal [55,56,57]. 

Several survey papers that provide an overview of the wideband spectrum sensing and compressive sensing are shown in Table 1 [53,54,56,68,69,70]. The authors of [53,54,56] reviewed the compressive sensing theory and discussed its applications in cognitive radio networks. In [61], the authors classified the wideband sensing techniques into two types, Nyquist wideband sensing and sub-Nyquist wideband sensing, and compared the advantages and disadvantages of each type. In [68], the authors provided an overview of specific narrowband sensing methods and discussed some of the associated challenges. These authors also provided a performance comparison between these techniques in terms of accuracy and complexity. The authors of [69] offered an overview of some of the wideband spectrum sensing techniques and classified them into three main categories: detection using a known sub-bandwidth, jointly estimating the boundaries and power density level of the sub-bands, and estimating the sub-band boundaries. 

In light of the recent research, this paper provides an in-depth survey of the most recent advances in spectrum sensing. Based on our analysis and evaluation of these techniques, we highlight the efficiency and limitations of these techniques as well as the challenges of implementing them. Then, we present case studies of methods describing how compressive sensing and machine learning principles can be incorporated into cognitive radio systems. We also discuss useful future research directions such as the need for fundamental research works related to security and energy efficiency in these networks. The reader may find that this survey covers some concepts dealt with in other surveys, but this is only to provide a better understanding of the issues related to spectrum sensing in cognitive radio networks. A distinctive feature of this survey is the inclusion of compressive sensing and machine learning theories. In addition, we describe how cognitive radio is unlocking the TV White Spaces spectrum.

This survey paper was designed and written in a way to help new researchers in the field to become familiar with spectrum sensing and yet to help advanced researchers to better understand issues related to state-of-the art techniques and undertake further research that helps to enable cognitive radio networks.

In short, the contributions of this paper can be summarized as follows:

- Classification of different spectrum sensing techniques
- Review of narrowband sensing techniques including traditional sensing techniques and machine learning-based sensing techniques.
- Analysis of the advantages and limitations of narrowband spectrum sensing techniques
- Review of Nyquist-based wideband spectrum sensing techniques
- Review of compressive sensing and its application in wideband spectrum sensing
- Analysis of the advantages and limitations of both wideband sensing techniques
- Discussion of how cognitive radio is unlocking TV White Spaces spectrum and challenges related to that.
- Discussion of the challenges and open issues involved in spectrum sensing and how cognitive radio technology can be used to solve radio spectrum access and interference management in future networks.

The remainder of this paper is organized as follows: Section 2 provides a classification of spectrum sensing techniques. Section 3 describes narrowband sensing techniques, analyzes them, and compares their performance. Section 4 reviews wideband spectrum sensing techniques and discusses the advantages and disadvantages of each approach. Section 5 discusses the TV Whites Spaces and how cognitive radio is applied to unlock this portion of the radio spectrum. Section 6 discusses several research challenges and future research directions. Finally, conclusions and future research works are drawn in Section 7.

## 2. Classification

**Figure 1:**
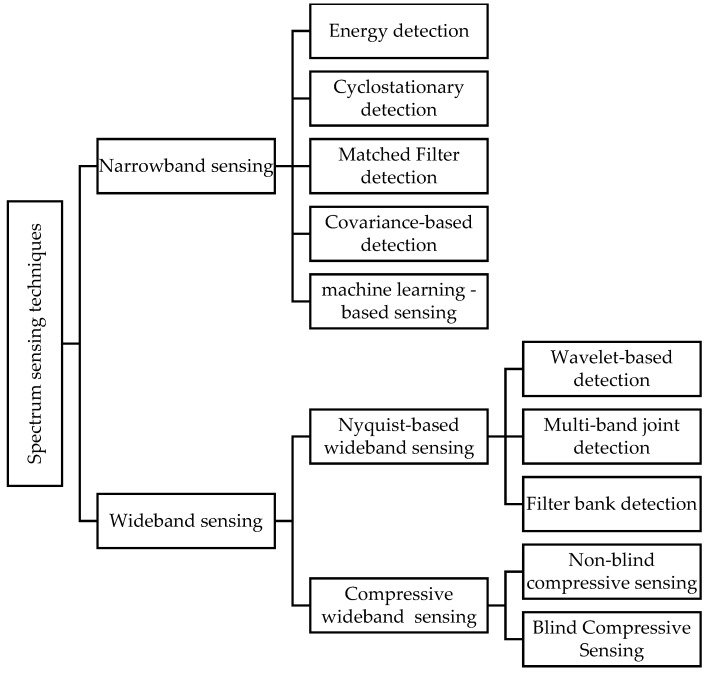
Classification of spectrum sensing techniques.

As shown in Figure 1, below, sensing techniques can be classified into two main categories: narrowband and wideband. Narrowband sensing techniques include energy detection [5,6,7,8,9,10,11,12,13,14,15,16,17,18,19,20], cyclostationary detection [21,22,23,24,25,26,27], matched filter sensing [28,29,30,31], covariance-based detection [32,33,34,35,36,37,38,39], and machine learning based sensing [40,41,42,43,44,45,46,47,48,49,50,51]. Of wideband spectrum-sensing’s two techniques, Nyquist-based uses analog-to-digital converters to sample the wideband signals at the Nyquist rate, which can result in a high sampling rate and power consumption. Techniques under this type include wavelet detection [71,72,73,74,75,76], multi-band joint detection [77,78], and filter bank based sensing [79,80,81,82]. Compressive sensing techniques sample signals below the Nyquist rate to reduce the high sampling rate [83,84,85,86,87,88,89]. Examples of these techniques include analog-to-information converter-based (AIC), two-step sensing [83,87], adaptive CS [88], and geo-location based CS [89]. These techniques can be classified into several types based on specific criteria. For example, compressive based sensing techniques can be classified into two types based on the estimation of the sparsity: those are based on sparsity estimation and those that perform blindly compressive sensing. 

## 3. Narrowband Spectrum Sensing

Based on the classification given in the previous section, we discuss each category and type including examples, the mathematical model, advantages, and disadvantages of each technique. 

### 3.1. Narrowband Sensing Techniques

Narrowband spectrum sensing techniques allow secondary users to decide on the presence or absence of the primary user over a frequency channel of interest. We assume that ${ℋ}_{0}$ indicates that the primary user signal is not present does not exist and ${ℋ}_{1}$ indicates that the primary user signal is present. The model of the received signal under these two assumptions, ${ℋ}_{0}$ and ${ℋ}_{1}$, can be expressed as:

(1) $${ℋ}_{0}:\;y\left(n\right)=\eta \left(n\right)$$

and:

(2) $${ℋ}_{1}:\;y\left(n\right)=s\;\left(\;n\right)+\eta \left(n\right)$$

where y(n) represents the received signal, η(n) represents a Gaussian white noise with a mean of 0 and a variance $\sigma^{2}$, and s(n) is the transmitted signal, and n denotes the sensing time.

The state ${ℋ}_{0}$ corresponds primary user absence and state ${ℋ}_{1}$ corresponds primary user presence. For the sensing decision, several of the previously mentioned spectrum sensing techniques can be used, including energy detection [5,6,7,8,9,10,11,12,13,14,15,16,17,18,19,20], cyclostationary detection [21,22,23,24,25,26,27], matched filter detection [28,29,30,31], covariance-based detection [32,33,34,35,36,37,38,39], and machine-learning based detection [40,41,42,43,44,45,46,47,48,49,50,51] which are discussed below. These techniques are often evaluated using the probabilities of false alarm and probability of detection. These two probabilities can be defined as

(3) $$P_{f}=p({ℋ}_{0}|\;{ℋ}_{1})$$

and:

(4) $$P_{d}=p({ℋ}_{0}|\;{ℋ}_{0})$$

#### 3.1.1. Energy Detection

**Figure 2:**
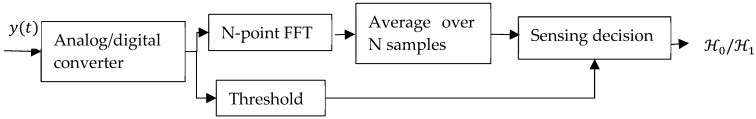
Block diagram of Energy Detection [5,6,7,8,9,10,11,12,13,14,15,16,17,18,19,20].

Energy detection [5,6,7,8,9,10,11,12,13,14,15,16,17,18,19,20] computes the energy of the samples and compares it to a threshold. If the energy is higher than this threshold, the primary user signal is considered present; otherwise, the primary user is considered absent. The concept, shown in Figure 2, calculates the energy of the samples as the squared magnitude of the FFT averaged over the number of samples N. This is given by:

(5) $$T_{\mathrm{ED}}=\frac{1}{N}\sum_{n=1}^{N}{\left(Y\left[n\right]\right)}^{2},$$

where N denotes the total number of received samples, and Y[n] denotes the nth received sample.

The result of this computation is then compared to a predefined threshold to obtain the sensing decision. If that energy is above the threshold, the primary user is considered present; otherwise, the primary user is considered absent. This is expressed mathematically as:

(6) $$T_{\mathrm{ED}}<\lambda_{\mathrm{ED}}\;\mathrm{Primary}\;\mathrm{User}\;\mathrm{absent},$$

and:

(7) $$T_{\mathrm{ED}}>\lambda_{\mathrm{ED}}\;\mathrm{Primary}\;\mathrm{User}\;\mathrm{present}$$

where $\lambda_{\mathrm{ED}}$ denotes the threshold that depends on the noise variance. The selection of the threshold, which can be static or dynamic, dramatically affects the detection performance.

Energy detection is a reasonably simple technique that does not require any prior knowledge of the signal characteristics. However, it cannot distinguish between the noise samples and the signal samples, which makes it subject to high uncertainty. In addition, it has a low detection performance for low signal-to-noise (SNR) values [21,26,28].

Several approaches have been proposed to improve the detection performance of energy detection using dynamic thresholds [6,16,17,20]. The authors of [3], for example, addressed the selection of the threshold by using a constant false alarm rate method that consists of bounding the probability of false alarm and then iteratively updating the value of the threshold to maximize the probability of detection. The authors of [16] proposed a discrete Fourier transform (DFT) filter bank method to dynamically select the threshold that minimizes the spectrum- sensing error in the presence of noise. The authors of [17] further proposed an adaptive threshold detection method based on an image binarization technique. This method dynamically estimates the threshold by taking the previous decisions and certain other parameters, including the targeted probabilities of detection and false alarm, SNR, and the number of samples into account. The authors of [19,20] proposed a double-threshold technique to deal with uncertainty where, if the energy of the samples is smaller than a certain threshold, then the band of interest is free, but if the energy of samples is higher than a second threshold, then the spectrum is occupied. Although this double-threshold algorithm decreases the collision probability, its detection performance is not acceptable for low SNR values, and its sensitivity to noise uncertainty is very high. In addition, its probability of misdetection is higher than with techniques that have only one threshold.

#### 3.1.2. Cyclostationary Feature Detection

Cyclostationary feature detection [21,22,23,24,25,26,27] relies on certain received signal features. Some statistics of the transmitted signal, such as modulation rate and carrier frequency, are periodic and perceived as cyclostationary features. Because the noise is stationary with no correlation, cyclostationary feature detection techniques can distinguish between the signal and noise by analyzing the spectral correlation function of the signal. 

The received signal y(t) is called cyclo-stationary if the mean and the autocorrelation of the signal are periodic. Mathematically, this is expressed as:

(8) $$m_{y}\left(t\right)=E\left[y\left(t\right)\right]=m_{y}\left(t+T_{0}\right),$$

(9) $$R_{y}\left(t,\tau \right)=R_{y}\left(t+T_{0},\tau \right),$$

where $T_{0}$ denotes the period of the signal y(t), E denotes the expectation operator, $R_{y}$ denotes the autocorrelation function of y(t), and τ denotes the time offset. The autocorrelation $R_{y}$ of the received signal y(t) is given by:

(10) $$R_{y}\left(\tau \right)=E\left[y\left(t+\tau \right)y^{*}\left(t-\tau \right)e^{j2\pi \alpha t}\right],$$

where E[·] denotes the expectation operator, and α denotes a cyclic frequency.

**Figure 3:**
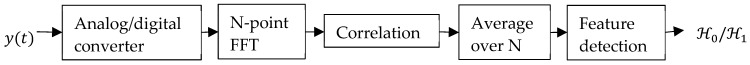
Block diagram of cyclostationary features based techniques [21,22,23,24,25,26,27].

Figure 3 shows this technique’s concept where the received analog signal is digitized using an analog-to-digital converter block, and its fast Fourier transform is computed by the N-point FFT block. These FFT values are correlated with themselves and then averaged over the number of samples N. The average of the outcome is then subjected to feature detection to obtain the sensing decision.

Cyclostationary techniques provide better detection performance than energy detection techniques. Moreover, their ability to differentiate between signals and noise allows these techniques to be less susceptible to noise uncertainty and hence have a lesser probability of false alarm compared to energy detection-based techniques. As for energy detection, the performance of the cyclostationary detection can be further enhanced by increasing the number of samples. However, this can result in an increase in sensing time and complexity as the length of the received signal increases. A balance between sensing time and performance detection must be found to reduce this complexity and still achieve an acceptable detection performance.

An improved version of this technique called correlation-based Euclidean distance has been proposed to reduce noise uncertainty [23]. It is based on the cyclic autocorrelation function, defined at lag l by:

(11) $$ACF\left(l\right)=\sum_{m=0}^{N-1}x\left(m\right)x^{*}\left(m-l\right),$$

where *N* is the number of samples, l is the lag in time required to produce the time-shifted version of the received sample x(m), and * represents the complex conjugate of the functions. 

If two successive values of the autocorrelation function of a signal are close to each other, then the signal is increasingly correlated. To determine the degree of correlation, the authors of [23] proposed a reference vector $ACF_{REF}$ that consists of the auto-correlated values of a signal that are strong enough to be sure of its presence. The correlation distance is computed as the difference between $ACF_{S}$, which consists of the auto correlated values of the received signal samples, and the reference vector, given by:

(12) $$d_{c}=\sqrt{\sum ACF_{REF}-ACF_{S}},$$

The correlation distance $d_{c}$ is then compared to a threshold γ to decide whether signal is present.

#### 3.1.3. Matched Filter Detection

**Figure 4:**
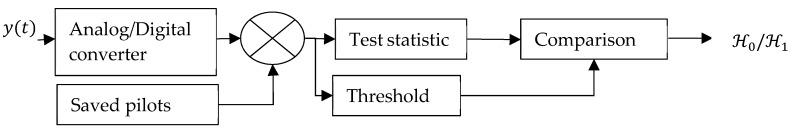
Block diagram of matched filter based spectrum sensing.

Matched filter sensing techniques [28,29,30,31] compare the received signal with pre-allocated and pilot samples captured from the same transmitter. These pilot samples are used to compute the test statistic, which is then compared to a threshold. If higher than the threshold, the signal is regarded as present. This process is shown in Figure 4.

The test statistic for the matched filter technique is given by:

(13) $$T_{\mathrm{MFD}}=\frac{1}{N}\overset{N}{\underset{n=1}{\sum }}y\left(n\right)x_{p}^{*}\left(n\right),$$

where N denotes the number of samples, y is the vector of samples, and $x_{p}$ are the pilot samples. This test statistic is then compared to a threshold to determine the sensing decision, such that:

(14) $$T_{\mathrm{MFD}}<\lambda_{\mathrm{MFD}}\;\mathrm{Primary}\;\mathrm{user}\;\mathrm{absent},$$

(15) $$T_{\mathrm{MFD}}>\lambda_{\mathrm{MFD}}\;\mathrm{Primary}\;\mathrm{user}\;\mathrm{present},$$

where $\lambda_{\mathrm{MFD}}$ denotes the threshold, which depends on the noise level present in the received signal. As for the previously mentioned techniques, the use of a static threshold can lead to less accurate results because of the noise uncertainty. This issue has motivated the authors of [25] to investigate the use of a dynamic selection of the threshold to enhance the detection performance of this sensing technique. Matched filter techniques are optimal in the sense that they require only a few samples to achieve good performance detection, but they are not very practical, as prior information about the primary user signal is not always available.

#### 3.1.4. Covariance-Based Detection

**Figure 5:**
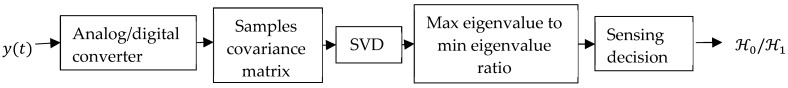
Block diagram of covariance-based based techniques.

Covariance-based detection techniques use sample covariance matrix of the received signal and singular value decomposition (SVD) to detect the presence of the primary user signal [32,33,34,35,36,37,38,39]. This is determined by evaluating the structure of the covariance matrix of the received signals. The signals from the primary user are correlated and can be differentiated from the noise. Using the singular value decomposition method the eigenvalues of this matrix can be determined. Then, the ratio between the maximum eigenvalue and minimum eigenvalue is calculated and compared with a threshold to decide between the two states, ${ℋ}_{0}$ and$\;{ℋ}_{1}$. The block diagram of the covariance-based detection is shown in Figure 5.

To compute the sample covariance matrix of the received signal, the following expression is used:

(16) $$R_{y}\left(N\right)=\frac{1}{N}\overset{L-2+\mathrm{Ns}}{\underset{n=L-1}{\sum }}\hat{y}\left(n\right){\hat{y}}^{†}\left(n\right),$$

where N is the number of collected samples.

The singular value decomposition operation applied on the matrix $R_{y}\left(N\right)$ gives the maximum and minimum eigenvalues of $R_{y}\left(N\right)$, that is $\lambda_{max}$ and $\lambda_{min}$. The test statistic, which is calculated as the ratio of maximum to the minimum eigenvalue, $\lambda_{max}/\lambda_{min}$, is then compared to a threshold to determine the sensing decision. Such that if the test statistic is below a threshold, the primary user is declared absent; otherwise the primary user is declared present. This can be expressed as:

(17) $$\lambda_{max}/\lambda_{min}<\gamma_{c}\;\mathrm{Primary}\;\mathrm{user}\;\mathrm{absent},$$

(18) $$T_{\mathrm{MFD}}>\gamma_{c}\;\mathrm{Primary}\;\mathrm{user}\;\mathrm{present},$$

where $\gamma_{c}$ is a pre-defined threshold.

#### 3.1.5. Machine Learning Based Spectrum Sensing

Machine learning has received increasing interest and has found application in many fields due to its ability to apply complex mathematical calculations to analyze and interpret patterns and structures in data, enabling learning, reasoning, and decision making. In the context of cognitive radio networks, several research papers related to machine learning for spectrum sensing have been published [42,43,44,45,46,47,48,49,50,51]. These machine learning-based sensing techniques aim at detecting the availability of frequency channels by formulating the process as a classification problem in which the classifier, supervised or unsupervised, has to decide between two states of each frequency channel: free or occupied. These classifiers use features, such as the energy statistic or probability vector, to determine the availability of channels.

Table 2 shows examples of works that apply machine learning for spectrum sensing to detect the availability of frequency channels [42,43,44,45,46,47,48,49,50,51]. For instance, the authors of [42,43] proposed a spectrum sensing model for cognitive radio based on both K-means and support vector machine. First, K-means is applied to discover primary users’ transmission patterns and statistics. Then, support vector machine (SVM) method is used to distinguish between two states: presence or absence of the primary user signal. The authors of [44] proposed a machine-learning model for compressive wideband spectrum sensing. Accurately estimating the sparsity allows the selection of the appropriate number of measurements for accurate recovery. Linear regression and support vector regression techniques are used to design a prediction technique to estimate the sparsity level of a wideband signal based on the occupancy of frequency channels. 

Machine learning has also been applied to perform cooperative spectrum sensing in cognitive radio networks. The existing works can be classified into two main categories. The technique in the first category uses two steps. In the first step, unsupervised machine learning techniques are used to analyze data and discover the primary user’s patterns. In the second step, supervised machine learning techniques are used to train the model with the data labeled in the first step. For instance, the authors of [42,43,46] proposed a two-step machine learning model for spectrum sensing. In the first step, the K-means algorithm is used to identify the state of the primary user’s presence. In the second step, support vector machine or other types of classifiers are used to attribute the new input data into one of the classes specified by the K-means method used in the first step. Techniques of the second category assume that the classes are known, and they are based on supervised machine learning to train models. The authors of [45,47,48], for example, used only one step in which supervised machine-learning classifiers: K-nearest neighbor, support vector machine, Naïve Bayes, and decision tree, are applied. In other works [42,45,47], the authors used different classifiers to train their models then select the best classifier for their models.

Selecting appropriate features for machine learning is critical to achieving high detection; several such features have been considered in previously mentioned papers. For example, the authors of [42,43,45,47,48] used energy statistic while the authors of [44] used occupancy and the authors of [46] used probability vector.

To evaluate these models, several evaluation metrics have been used. The authors of [42] used the probabilities of detection and false alarm as well as the training duration and classification delay. The authors of [43] used discrimination probability in addition to probabilities of detection and false alarm. The authors of [44] used the probabilities of miss-detection and false alarm. The authors of [45] used the accuracy and probabilities of detection and false alarm. The authors of [46] used throughput while the authors of [48] used error rate.

### 3.2. Performance Comparison of the Narrowband Sensing Techniques

Table 3, below, compares the aforementioned narrowband sensing techniques. The first, energy detection, does not require any prior knowledge about the signal characteristics, which makes this technique simple and easy to implement. Its limitations include the inability to distinguish between the signal and noise. It also has a low detection performance at low SNR values, and it is more susceptible to noise uncertainty. Dynamic selection of the threshold and increasing the sample number can enhance the performance of this sensing technique, but at the cost of increasing the sensing time.

Unlike energy detection, cyclostationary detection is robust against noise as it can readily distinguish between signal and noise. Likewise, this technique also requires a high number of samples to achieve a better detection performance, which increases the sensing time. Moreover, this technique is especially complex for large signals.

Matched filter sensing is the optimal technique for sensing since it needs only a few samples to achieve a good detection performance. Its main drawback, however, is that prior knowledge of the signal is required, which may not always be available. In addition, this technique is unreliable in the presence of primary user emulation attacks where a malicious node mimics the signal of the primary user, making this technique less useful in practical scenarios. Between the three, cyclostationary features detection strikes a good balance between complexity, performance, and practicability.

More sophisticated detection techniques use the sample covariance matrix. These techniques are as accurate as cyclostationary detection and matched filter, but they have high computational complexity. Computation of the sample covariance matrix and its singular value decomposition are the two major sources of the computational complexity. The complexity of these techniques makes them not suitable for real-time applications. 

Machine learning can be a good approach compared to the previous narrowband sensing techniques as it does not require to set any threshold. It can minimize the detection time, but it is more complex than the other sensing techniques. Another issue with machine learning based sensing technique is the dataset to perform the classification. In addition, the training of the machine learning models has to be updated in a very fast changing environment, which adds more complexity to the sensing process. Furthermore, the feature selection is also another task that has to be well thought.

## 4. Wideband Spectrum Sensing

The next generation of communication systems requires high data rates and therefore high bandwidth. From this perspective, secondary users need to sense wide frequency ranges of the radio spectrum to find the best available channels. As a result, several types of approaches have been proposed to perform wideband spectrum sensing [61]. Most approaches divide the wideband spectrum into several narrow bands and perform sensing sequentially, a process that increases sensing time as they scan one band at a time. Another solution is to sense the narrow bands in parallel by using multiple sensors and performing joint detection. This approach is also unpractical because of the hardware cost and the fact that sensor synchronization is required. As an alternative, researchers have investigated compressive sensing techniques to reduce the number of samples, reducing the sensing delay. In the following discussion, we review the most relevant sensing techniques from both types, Nyquist and sub-Nyquist based, present their advantages and disadvantages, and discuss the efficiency of each type.

### 4.1. Nyquist Wideband Spectrum Sensing

Conventional wideband spectrum sensing techniques use standard ADC converters operating at the Nyquist rate to sample the wideband signal. Examples include wavelet, multi-band joint detection, and filter bank sensing. In the following sections, a review of these techniques is given as well as the advantages and limitations of each approach.

#### 4.1.1. Wavelet Detection

**Figure 6:**
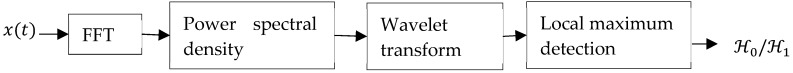
Block diagram of wavelet based spectrum sensing [71,72,73,74,75,76].

Suppose that the received signal occupies M spectrum bands whose frequency locations and power spectral density are unknown and should be detected. A wavelet approach for spectrum sensing is considered to be an edge detection of the occupied band since wavelet transforms can characterize these edges. As shown in Figure 6, the wideband signal for this approach is decomposed into elementary building blocks of sub-bands, characterized by local irregularities in the frequency domain. The wavelet transform is then applied to detect the local spectral irregular structure, which carries important information about the frequency locations and power spectral densities of the sub-bands [71,72,73,74,75,76].

Dividing each band into many channels and then sensing channel-by-channel sequentially introduces significant latency. One possible way to reduce this latency is to jointly sense multiple bands using an RF front-end with a bank of narrow band-pass filters.

#### 4.1.2. Multi-Band Joint Detection

**Figure 7:**
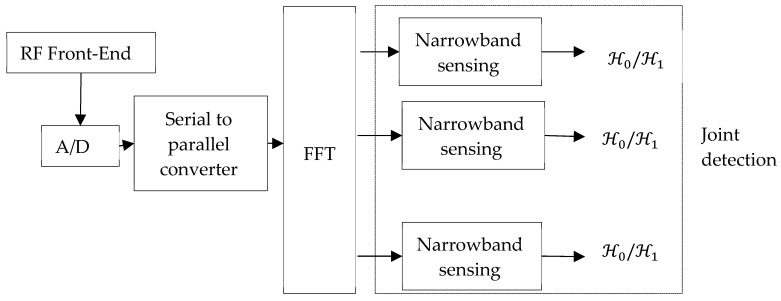
Block diagram of multiband joint detection [77,78].

As shown in Figure 7, multi-band joint detection techniques sense the presence or the absence of a primary user over multiple frequency bands rather than one band at a time [77,78]. Given a cognitive radio device that is sensing K narrow bands, for each band a sensing technique such as the energy of the received signal over an interval of N samples is calculated and then compared to a threshold to decide whether the band is occupied or not. For each band, the probabilities of detection and false alarm are functions of the threshold. The objective of multi-band joint detection is thus to find the optimal threshold vector that increases the probability of detection and decreases the probability of any false alarm for all the bands jointly by solving an optimization problem.

#### 4.1.3. Filter Bank-Based Sensing

**Figure 8:**
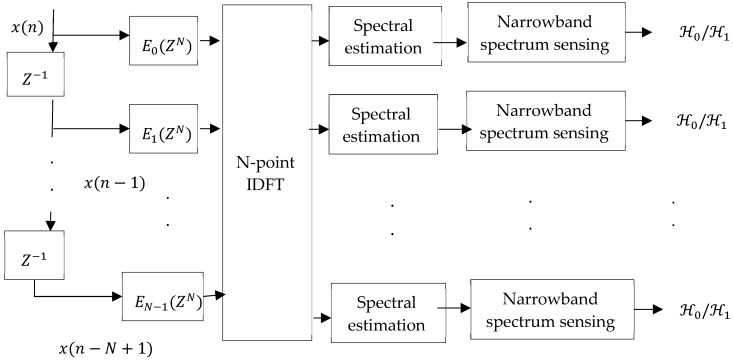
Block diagram of filter bank based spectrum sensing [79,80,81,82].

Wideband spectrum sensing usually involves estimation of power spectral density (PSD). One way to estimate this function is to use the filter bank approach [79,80,81,82] which can enable an efficient implementation of band-pass filters by using a poly-phase decomposition of the prototype filter [79]. The concept of filter bank analysis, as shown in Figure 8, consists of an array of band-pass filters. Each filter is the frequency-shifted copy of a low-pass filter. After the output of each filter bank, an energy detector is placed to determine whether the PU is active or is not.

### 4.2. Sub-Nyquist Wideband Sensing

Each of the Nyquist-based sensing techniques has limitations, such as a high sampling rate and power consumption, are not acceptable for the next generation of communication systems. Therefore, several compressive-based techniques have been proposed to mitigate the sampling rate issues. The application these methods in the context of wideband spectrum sensing is motivated by the scarcity of the spectrum and the sparsity of the wideband signal in the frequency domain.

#### 4.2.1. Compressive Sensing-based Wideband Spectrum Sensing

Compressive sensing can be classified into two main categories: multi-bit compressive sensing and one-bit compressive sensing. In the following, we describe each of these categories.

##### Multi-Bit Compressive Sensing Mathematical Model

**Figure 9:**
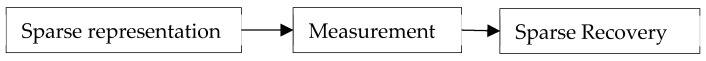
Block diagram of compressive sensing [53,54,56,67].

Compressive wideband spectrum sensing has recently been the subject of many research studies [53,54,56]. The application of compressive sensing in this context is made possible because most of the frequency channels are free, which makes a wideband signal sparse in the frequency domain [57,58,59,60]. Compressive sensing is defined as a paradigm that recovers the sparse signal from a few measurements [53,54,56,67]. The foundations upon which this paradigm rests are the sparsity and incoherence. As shown in Figure 9, compressive sensing involves three main processes: sparse representation, measurement, and sparse recovery. The sparse representation consists in projecting the signal on a suitable basis to make the signal sparse because compressive sensing applies only to sparse signals. Examples of sparse representation techniques include fast Fourier transform (FFT), discrete Fourier transform (DFT), and discrete cosine transform (DCT). The measurement process takes only a few measurements from the sparse signal by multiplying this signal by a measurement matrix, recovering the original sparse signal. In the following discussion, we describe the mathematical model of each process.

Given a signal x with a number of samples N, this signal is assumed to be sparse in a given domain and its sparsity level K satisfies K<<N. If the signal is not sparse in the given domain, a projection of this signal x on a suitable basis can make it sparse. This signal s is given by:

(19) s=φ∗x,

where φ is sthe parse basis and if the signal x is sparse then this basis is the $=I_{N}$.

For the remainder of this paper, we consider that the signal x is sparse without loss of generality.

The measurement is taken by multiplying the sparse signal by an M×N measurement matrix, Φ, where M is the number of measurements that depend on the sparsity level of the sparse signal, the measurement matrix, and the recovery method. The vector of measurement y is then given by:

(20) y=Φ∗x,

The measurement matrix, Φ, must satisfy the restricted isometry property or have a small mutual coherence. The coherence denotes the maximum correlation between any two columns of the measurement matrix. Given an M×N normalized matrix Φ whose column vectors are $\Phi_{j}\;\left(j=1,\ldots ,\;N\right)$, the mutual coherence constant, *c*, is given by:

(21) $$c=\underset{j\neq k}{\max}\left|\Phi_{i},\Phi_{i}\right|,$$

Φ must have a low coherence to guarantee recovery with small error.

A matrix is said to satisfy the restricted isometry property if there is a constant $\varepsilon_{k}$ such that:

(22) $$\left(1-\varepsilon_{k}\right){\left\|x\right\|}_{2}^{2}\leq \Phi *{\left\|x\right\|}_{2}^{2}\leq \left(1+\varepsilon_{k}\right){\left\|x\right\|}_{2}^{2},$$

where ${\left\|.\right\|}_{2}$ is the $l_{2}$-norm, $\varepsilon_{k}\in \left[0,1\right]$ is the restricted isometry constant (RIC) of the matrix Φ, and x is k-sparse signal.

Over the last decade, several measurement matrices have been proposed which can be classified into two primary categories: random and deterministic [67]. Random matrices are generated by identical or independent distributions, including Gaussian, Bernoulli, and Uniform matrices [90]. In the deterministic category, measurements are generated following a given structure. Examples include circulant, Toeplitz, and chirp sensing [54,67].

The sparse recovery process, which is an undetermined system of linear equations, recovers the original signal from few measurements [56]. This sparse recovery process can be stated:

(23) $$\min{\left\|x\right\|}_{0}\;\mathrm{subject}\;\mathrm{to}\;y=\Phi *x,$$

where ${\left\|.\right\|}_{0}$ is the $l_{0}$-norm, x is the sparse signal, Φ is the measurement matrix, and y is the vector of measurements. Because solving this problem is challenging, its convex relaxation is considered, replacing the $l_{0}$-norm by $l_{1}$-norm. The sparse recovery problem can then be re-written as:

(24) $$\min{\left\|x\right\|}_{1}\;\mathrm{subject}\;\mathrm{to}\;y=\Phi *x,$$

where ${\left\|.\right\|}_{1}$ denotes the $l_{1}$-norm.

To solve this problem, several recovery techniques have been proposed, which may be classified into three categories: convex and relaxation, greedy, and Bayesian. Convex and relaxation solves the sparse recovery problem using optimization algorithms such as the gradient descent [91]. Greedy methods build the solution iteratively to reduce processing time. Examples include orthogonal matching pursuit and its derivatives [92,93,94,95]. Finally, Bayesian recovery techniques use Bayesian models to define the prior distribution for the sparse signal and find the optimal solution. Examples include Bayesian via fast-Laplace prior, Bayesian via relevance vector machine, and Bayesian via belief propagation [96,97,98].

##### One-Bit Compressive Sensing

For most communication systems, a quantization step is required prior to the signal processing step which leads to multi-level quantization errors. The quantization is typically modeled as a measurement noise with limited energy [99]. Thus, this quantization can be formulated as:

(25) $$y_{i}=Q\left(\Phi *x\right)=\Phi *x+n,$$

where Q(.) denotes the quantizer and *n* is a limited-energy measurement noise.

To minimize the multi-level quantization errors, one-bit compressive sensing has been proposed recently as a promising solution [99,100,101,102,103,104,105,106,107,108]. This technique performs 1-bit quantization using a quantizer most often implemented as a comparator to a level l, which is often zero.

**Figure 10:**
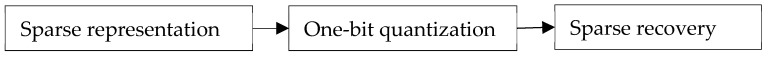
Theoretical framework of one-bit compressive sensing [92,99,100,101,102,103,104,105].

The framework of one-bit compressive sensing, shown in Figure 10, consists of sparse representation, one-bit quantization, and sparse recovery. One-bit compressive sensing preserves the sign information of the measurements, reducing the hardware cost.

The vector of measurement in this technique is obtained from the measurement system:

(26) $$y_{i}=sign\;\left({\left(\Phi *x\right)}_{i}-l\right))=sign\;\left({\left(\Phi *x\right)}_{i}\right)$$

where sign(.) denotes an operator that performs an element wise sign function on a vector, Φ denotes the measurement matrix, and x denotes the sparse signal.

The product of each quantized measurement with the measurement is positive. This property, called the consistency principle, is given by:

(27) $$y_{i}sign\;\left({\left(\Phi *x\right)}_{i}\right)\geq 0$$

Thus, in a compact version, the vector of measurement can be re-written as:

(28) y=sign (Φ∗x)

where Φ is the measurement matrix, x is the sparse signal, and sign(.) is the 1-bit quantization function applied element wise to the vector Φ∗x.

Thus, the equation (Equation (21)) can be re-written as:

(29) YΦx≥0

where Y=diag(y).

Recovery algorithms for one-bit compressive sensing are different from the classical recovery algorithms for multi-bit compressive sensing. They can be grouped into three categories: regularizer, penalty-based, and Bayesian-based. Regularizer techniques add a regularization term to the optimization model to quantify the sign violation [99,100,101,102,103,104], penalty-based algorithms minimize a penalty function to recover the sparse signal [102,103,104,105], and Bayesian-based techniques underline a probability distribution functions to the noise vector and the sparse signal and then maximize the likelihood function to find the hyper-parameters to recover the original signal [107,108].

#### 4.2.2. Application of Compressive Sensing in Wideband Spectrum Sensing

In the context of wideband spectrum sensing, we consider a wideband signal divided into N sub-bands. Since most bands in the signal are vacant, the received signal can be expressed as:

(30) $$Y\left(f\right)=\sum_{n\in S}D_{h}.x_{n}\left(f\right)+w\left(f\right),$$

where $D_{h}$ is a diagonal N×N channel gain matrix, $x_{n}\left(f\right)$ is the signal of the primary user, w(f) is an additive noise, and S is the set of occupied channels.

The received signal is sensed using a measurement matrix to acquire the compressed samples [69]. This vector of measurements is given by:

(31) $$z_{f}={\Phi y}_{f},$$

where $z_{f}$ is M × 1 measurement vector, Φ is an M×N sensing matrix, $y_{f}$ is the projection of the received signal in the frequency domain, and M is the number of measurements, which is dependent on the sparsity level given by the cardinality of the set of occupied channels S. The signal is then reconstructed from this set of measurements using one of the aforementioned sparse recovery techniques. Once the recovery is performed, the sensing decision can be obtained directly from the recovered signal by finding the location of the zero and non-zero components of the signal as the locations can be applied to obtain the detection decision.

**Figure 11:**
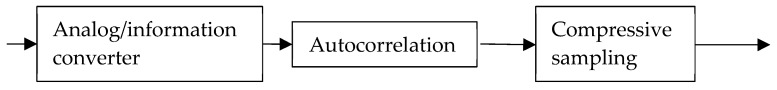
Concept of compressive sensing as introduced in [64].

The concept of compressive wideband spectrum sensing was first introduced by Tian et al. [64]. These authors proposed a scheme, shown in Figure 11, based on an analog-to-information converter (AIC) that transforms the received analog signal at a receiver into a digital signal. It consists of pseudo-random number generators that produce the partial measurements. An autocorrelation of the compressed measurements is then used to reconstruct an estimate of the wideband signal. This approach reduces the high sampling rate since it does not use a high number of samples. However, it does have some drawbacks, such as high computational complexity because of the large size of the measurement matrix and the performance of the AIC model that can be easily affected by design imperfections.

After the introduction of the concept, multiple approaches have been proposed which can be classified into two subtypes. The first type, non-blind compressive wideband sensing, requires the estimation of the sparsity level of the wideband signal to perform the measurement while the second type, blind compressive sensing, does not require any prior knowledge of the sparsity level of the wideband signal. In the following, we describe the concept of each type.

a. Non-Blind Compressive Wideband Sensing Techniques

Non-blind compressive wideband sensing techniques determine the parameters of the wideband signal such as its sparsity, or its occupancy, the number of measurements required before performing the measurement process. The sparsity level of the wideband signal can be determined by estimating the number of occupied frequency channels within a band of interest. Then, the number of measurements that can be determined, which is a function of the sparsity level, measurement matrix, and the recovery algorithm.

Several techniques have been proposed to determine these parameters [83,84,85,86,87]. For instance, in [83,87], the authors proposed a concept for estimating the wideband signal sparsity order. This technique uses a small number of compressed samples, $M_{e}$, for which the signal is recovered. Once $M_{e}$ is determined, a statistical learning methodology is used to determine the number of samples $M_{r}$ via the data fitting to be acquired. It then reconstructs the wideband signal from the compressed samples and makes the spectrum sensing decision. In [84,87], the authors used a two-step compressive sensing model for minimizing the sampling rates. The first step of this scheme estimates the sparsity level and adjusts the number of measurements to be used for the sampling in the second step. Then the signal is reconstructed from these few measurements and energy detection is applied to obtain the sensing decision. In [84], the authors proposed a technique that consists of selecting the number of measurements based on the recovery error. The number of compressed measurements in each time slot is adjusted interactively based on the recovery error for the previous time slot. If the recovery error in a given time slot is higher, then the number of measurements for the next time slot is increased, otherwise, the measurement number is decreased. This process is repeated until an accurate number of measurements is found. This methodology gives good performance in term of the recovery error, however, the interactive process introduces more complexity for the model instead of reducing it.

To reduce this complexity, the authors of proposed another technique that can estimate the sparsity level of the wideband signal and thus reduce the number of measurements. The proposed framework combines compressive spectrum sensing with a geo-location database [88]. This database provides the prior knowledge necessary to estimate the sparsity level of the signal by returning a list of available frequency channels. The sensing nodes then use this information to determine the accurate number of measurements that leads to a small recovery error. However, if this database is not updated in real time, it can give false information, which can result in inaccurate sensing decisions.

b. Blind Compressive Wideband Spectrum-Sensing Techniques

An estimation of the parameters of compressive sensing, such as sparsity level and the number of measurements, is often required to minimize the recovery error and enhance detection performance or consult an external entity to exchange information about the occupancy of the wideband spectrum. However, estimation of sparsity adds more complexity to the sensing process because doing so often requires more traffic exchanges between the sensing nodes and an external database or the use of one additional block to estimate that sparsity. Several techniques have been proposed to address this issue by introducing methods that do not require any prior knowledge of the sparsity level [85]. For example, the authors of [65] suggested a discrete cosine transform to concentrate the energy of the signal in a few samples of the DCT domain signal. According to the authors, the proposed algorithm does not require prior knowledge or estimation of the PU signal sparsity. Another example of blind techniques was proposed by the authors of [85]. This technique blindly detects the locations of active frequency channels. It requires prior information about the upper bound on the total number of active sub-bands and the maximum bandwidth of the active sub-bands and directly estimates the power spectrum of the entire frequency band using the coset samples before performing the sensing. The main drawback of conventional multi-coset approaches is that they require the number of active bands to be known. Also, the number of branches cannot be changed after the implementation.

#### 4.2.3. Comparison of Wideband Spectrum Sensing Approaches

To evaluate the performance of wideband spectrum sensing techniques, several evaluation metrics can be used including probability of detection, probability of miss-detection, probability of false alarm, and sensing time. Probability of detection is an important metric that measures the likelihood that a sensing technique detects that the primary user signal is present when that signal is present. Probability of miss-detection measures the likelihood that the sensing technique detects that the primary user signal is absent while it is absent. A method with a high probability of miss-detection can make secondary users transmit in an occupied channel thereby disrupting the communication of primary users. Probability of false alarm measures the likelihood that the sensing technique detects that the primary user signal is present while it is absent. A method with a high probability of false alarm decreases the chance that secondary users can access free frequency channels. Sensing time depends on two main parameters: the number of channels sensed and the number of samples collected for each channel. As discussed earlier, reducing the number of collected samples can reduce the processing time, but at the cost of the detection performance. A tradeoff between the size of the samples and the detection performance should be carefully considered.

Using these evaluation metrics, Table 4 summarizes the advantages and disadvantages of the relevant wideband sensing techniques. As shown in this table, methods using the Nyquist rate, such as wavelet, multi-band joint detection, and filter bank require high sampling rates, can result in a high sensing time and energy consumption. Compressive sensing techniques reduce the sampling rate, sensing time, and energy consumption. However, these techniques have reduced performances compared to the Nyquist-based sensing techniques. In addition, compressive sensing techniques require estimating the sparsity of the wideband signal to determine the number of measurements to be used. Techniques that do the estimation dynamically minimize the recovery error at the expense of higher complexity. When applying the sensing technique directly, the vector of measurements can reduce the complexity of compressive sensing. This complexity can be reduced even more if these techniques do not require prior knowledge of sparsity estimation.

Signal recovery is yet another way to evaluate wideband sensing techniques. The aforementioned techniques [90,91,92,93,94,95,96,97,98] perform the recovery of the signal after the process of measurement. Other authors proposed to use compressive sensing without recovery. For instance, the authors of [71,72,73] stated that the process of recovery introduces greater complexity to the system, and suggest not recovering the wideband signal as the objective of spectrum sensing is to determine the locations of the active frequency channels. The author of [71] proposed an algorithm that enables the detection of the primary user signal directly from the compressed measurements without using the recovery process because the measurement matrix was designed to preserve the energy of the PU signal in only a few samples. However, approaches that do not perform the recovery can be applied only when acquiring a few measurements for each frequency band and all channels are sensed one-by-one. In contrast, if we consider the wideband signal as a sparse signal in the frequency domain, and we sense only a few frequency channels, if the recovery is not performed, then deciding on the occupancy of a channels not sensed can be challenging.

## 5. Unlocking TV White Spaces via Cognitive Radio

Television stations often operate on the same or adjacent frequency channels. To avoid interference, television stations often operate in geographically separate areas. In addition, in areas with low population density, not all television channels are utilized. These unused channels TV white spaces (TVWS), represent a valuable opportunity for rural connectivity. As digital TV has higher spectrum efficiency compared to analog TV, the trends of switching over from analog to digital TV have freed up more channels in the bands of TVWS [109,110,111,112,113,114,115,116,117,118,119]. Spectrum sharing through the TVWS spectrum is an important topic as it is the first step toward an efficient use of the spectrum in an opportunistic and dynamic manner through cognitive radio technology.

In 2010, the Federal Communications Commission (FCC) adopted some final rules to allow unlicensed radio transmitters to operate in the white spaces [112,113]. This decision made a significant portion of the spectrum available for new and innovative products and services. The TVWS has superior propagation characteristics as it includes the VHF and UHF frequency bands. This results in longer communication distance and better penetration through obstacles, such as building, trees, and rain, etc. These distinctive characteristics of TVWS make it an attractive option to the next generation networks and devices.

There are several rules that were adopted to unlock the TVWS spectrum. One of these rules aims to prevent interference to licensed users and other services by using a geo-location capability of the white spaces devices combined with White Space DataBase (WSDB) to identify available TV channels at specific locations [109,110,111,112,113]. The databases are established and administered by parties selected by the FCC. In other words, this rule obviated the spectrum sensing as the mechanism for the unlicensed users to determine the TVWS holes. Instead, unlicensed users are required to periodically access to a geo-located database service for acquiring the spectrum availability, with a fixed timeframe, referred to as database access period. The rules also specify that the spectrum availability information consists of a list of channels within which the unlicensed users are allowed to operate and for each channel the duration of such allowed operation.

There are several challenges related to unlocking the TV white space. These include the design of the optimal database access strategy that respects the existing rulings, maximizing the expected TV White Space communication, and interference management. These have received an increasing interest from researchers since the regulatory bodies have approved the dynamic access to TVWS. Further, IEEE started actively working on defining several new standards aiming at enabling TVWS communications, such as IEEE 802.22, IEEE 802.11af to allow Wi-Fi to operate in TVWS, IEEE 802.15.4m to allow machine-to-machine communication.

Several approaches were proposed to enable the use of the TVWS. For instance, the authors of [115] proposed to maximize the achievable data rate of an arbitrary Neighborhood Area Networks (NAN) subject to interference caused by NANs located within the same geographical area and authorized to use the same TVWS channel. For this, they proposed to allow the Gateway to sense the TVWS channels declared available from incumbents by the WSDB to discover the presence of an interfering NAN. If the sensing declares the TVWS channel as idle, the Gateway can transmit over such a channel. Otherwise, the Gateway uses the ISM channel. The authors of [116] developed a theoretical framework for the optimal deployment of multiple closely-located NANs over the TVWS spectrum. Specifically, they derived through closed-form expressions the optimal values for both the number of NANs and their coverage, i.e., the maximum values assuring the given collision constraint. Such closed-form expressions show a non-linear relationship between parameters such as the number of NANs, their traffic demand, the number of home area network (HAN) gateways and their transmission ranges.

In order to minimize the chance of harmful interference, the authors of [117] proposed a sensing technique based on the Grassmann covariance matrix (GCM) to sense the TV White Spaces. A new test statistic is defined using Binet–Cauchy metric. The authors showed that this test statistic is a valid detector. The only problem of this technique is its high complexity coming from the sample covariance matrix and the signal value decomposition operation. Further complexity reduction is required to make this technique suitable for real-time hardware implementation [117].

## 6. Challenges and Future Research Directions

In order to minimize the impact of harmful interference, spectrum sensing techniques have to have a high probability of detection and a low probability of false alarm. However, existing narrowband sensing techniques have several limitations. For instance, Energy detection cannot detect weak signals which are below thermal noise. In addition, advanced techniques such autocorrelation and cyclostationary feature require considerable processing power which is not desirable in portable devices. More sophisticated techniques such as Eigen-value based and sample covariance matrix have been investigated in TVWS and can achieve a good probability of detection, but they are far more complex than cyclostationary detection. Further investigation of complexity reduction is required to make these techniques suitable for real-time hardware implementation.

Wideband spectrum sensing requires high sampling rates, high-resolution A/D converters, and high-speed signal processors. Such requirements, the design of efficient, reliable, and accurate wideband spectrum sensing combined with low complexity and low computation cost is challenging. Despite the important advances already made in wideband spectrum sensing, this area still presents several challenges. These challenges are mainly the estimation of the sparsity level, selection of the number of measurements, and noise uncertainty [120,121,122,123,124,125,126,127,128,129,130].

Estimation of the sparsity level of the wideband signal is important, as it is used as prior knowledge for selecting the appropriate number of measurements. However, in practice it is difficult to keep acquiring this prior knowledge in a very high dynamic wireless environment. Moreover, additional processes to estimate the sparsity of this wideband signal can increase the complexity of the compressive wideband sensing, i.e., sensing time. Future cognitive radio systems will have to perform compressive wideband sensing with unknown sparsity level. Thus, a challenging task will be to develop blind sub-Nyquist wideband sensing techniques where no prior knowledge about the sparsity of the wideband signal is needed to perform the spectral reconstruction.

Selecting the number of measurements is also challenging. This number depends on the sparsity level of the wideband signal, which is variable. Some authors proposed adapting the number of measurements using a fixed sparsity level. However, in practice the sparsity level of the wideband signal varies over time and is difficult to estimate. Thus, there is a need for wideband sensing techniques that intelligently select an appropriate number of measurements without any prior knowledge of the sparsity level.

Another challenge is how to handle noise uncertainty [55,128,130]. Most of the compressive wideband spectrum sensing techniques use a static threshold, which depends on the level of noise. Since this noise is uncertain, these sensing techniques are not accurate. A few research papers have proposed techniques to deal with uncertainty, including those using probabilistic models [130]. Measurement of the noise is then needed to enhance the detection performance of the sensing techniques.

Compressive sensing does reduce the time of wideband spectrum detection, but the recovery time is high in the case of large-scale problems. To minimize this time, some authors proposed performing compressive sensing without the recovery process. However, no work has yet been done to evaluate techniques both with and without this recovery process. Investigation of the impact of skipping the recovery process on the detection performance is needed.

Another challenge is related to the sensitivity of wideband spectrum sensing techniques. Next generation communication systems are expected to operate at very low SNR values where compressive sensing techniques are usually inaccurate. Very little research has investigated wideband spectrum sensing efficiency at very low SNR although this is a promising area of research that ought to be considered as a possible future direction [5,6,7,8,9,10,11,12,13,14,15,16,17,18,19,20,131,132,133].

There is also a need for research that covers the physical implementation of wideband sensing techniques. Most techniques covered in this paper were validated only through simulations, with a few implemented in software defined radio (SDR) units [128,129]. In [128], the authors proposed a method using energy detection and autocorrelation. This technique was implemented using SDR units to perform spectrum sensing of bands ranging from 825 MHz to 5.8 GHz. In [129], the authors used SDR units to scan TV bands. Likewise, very few authors implemented compressive sensing techniques. The authors of [129] tested wideband compressive spectrum sensing on real-time signals collected by the CRFS RFeye node to monitor the radio spectrum from 10 MHz to 6 GHz. Thus, more work on the implementation of compressive wideband spectrum sensing techniques using SDR units still needs to be done to evaluate the proposed methods in real-world scenarios.

Because of its low complexity and computational cost, one-bit compressive sensing has been proposed as a way to overcome the limitations of classical compressive sensing. A comparison between the performances of one-bit compressive sensing with conventional compressive sensing has been investigated in [104] through simulations. The results show that 1-bit compressive sensing outperforms multi-bit compressive sensing techniques. However, the application of one-bit compressive sensing for wideband spectrum sensing is still in its infancy, and this method’s efficiency still needs to be investigated and compared to multi-bit compressive sensing in real-world scenarios.

Multiple-input multiple-output (MIMO) and beamforming technologies can also improve the spectrum sensing performance. MIMO is regarded as one of the more promising technologies for 5G wireless communication systems and a prospect mechanism for future dynamic spectrum access if combined with beamforming and cognitive radio technologies [132,133]. MIMO systems utilize the spatial multiplexing gain to achieve a high spectral efficiency. A MIMO system is composed of two main elements: a transmitter and receiver. Each element is comprised of multiple antennas. The received signal at each antenna is sampled by an individual ADC and all the received signals by the multiple antennas are processed and the signal with the best response is used by the sensing technique to determine the availability of the frequency channel. MIMO systems are believed to increase the probability of detection and decrease both the probabilities of miss-detection and false alarm. For sparse signals, the authors of [132,133] proposed compressive sensing schemes for MIMO systems. In contrast to a traditional MIMO system, a MIMO based compressive sensing uses a single ADC thereby reducing the hardware cost and energy consumption.

Another challenge is that the detection of a primary user signal is always compromised by shadowing, fading effects, and noise uncertainty. To mitigate these effects, cooperative spectrum sensing has been proposed as a viable solution to improve the detection rate by exploiting spatial diversity [134,135,136,137,138,139,140,141,142,143,144,145,146,147,148,149,150,151,152,153,154,155,156,157]. The cooperating nodes perform narrowband sensing using either Nyquist or sub-Nyquist-based techniques. The existing research has mostly focused on the centralized cooperative model in which the sensing nodes report their sensing results to the fusion center via a common control channel using soft combining or hard decision approaches. In the case of soft combining, the fusion center combines the samples using techniques like equal gain combining, maximal ratio combining [138], and linear rule [139]. In the case of a hard decision, the fusion center uses techniques such as AND-rule [139,140], OR-rule [141], K-out-of-N rules [142,143,144], or machine learning [145,146]. Cooperative sensing can also be performed in a distributed manner in which the secondary users collect reports from their neighbors and make the decision individually [147,148,149,150]. 

To reduce incurred overhead, techniques on collaborative spectrum sensing have been proposed, including a cooperative scheme guided by game theoretical models [149], random matrix theory [150], cluster-based cooperative spectrum sensing [151], and distributed rule-regulated [152]. More recently, applications of compressive sensing for cooperative spectrum sensing have emerged to release sensing nodes from sending and the fusion center from gathering an excessively large number of reports [153,154,155,156,157]. Some other research work focused recently on increasing the performance of cooperative spectrum sensing considering the mobility effect in cooperative sensing [158,159]. For instance, the authors of [158] proposed a solution for the problem of uncorrelated user selection in mobile cognitive radio ad hoc networks. Specifically, a fully distributed user selection algorithm is developed by adaptively selecting uncorrelated cognitive radio users. Such algorithm is able to account for dynamic changes in the network topology and in the channel conditions. The authors of [159] proposed a solution for tuning the sensing time parameters, i.e., the sensing time and the transmission time, according to the primary user network dynamics. Despite the advances made, cooperative spectrum sensing has several limitations, especially in scenarios under attacks, such as spectrum sensing data falsification where malicious nodes report false sensing data to mislead the fusion center of the neighbors. These attacks can significantly degrade the performance of wireless networks. Therefore, there is a clear need for more secure cooperative sensing techniques.

Detection of primary signals can be also compromised by attacks. The challenge is to detect and cope with attacks that target the sensing process of the cognitive radio systems such as the primary user emulation [160] and spectrum sensing data falsification attacks [161]. In primary user emulation, a malicious node emulates the characteristics of the primary user signal to prevent other secondary users from using the available channels. In spectrum sensing data falsification, a malicious user sends false reports to the fusion center or to other users to mislead them about the availability of the frequency channels. Such attacks prompt a strong need for effective low energy, secure schemes to guarantee a fair spectrum sharing among different entities [162]. Overall, the security aspect should also be addressed with spectral efficiency and energy efficiency as a single objective. Security and energy efficiency are two aspects that have been overlooked by researchers.

## 7. Conclusions

Given that billions of wireless devices are expected to be connected to the Internet by 2020, it is likely that accessing the radio spectrum will be a big challenge. This problem is one of the most urgent issues that has yet to be addressed. Cognitive radio technology has the potential to address challenges associated with spectrum access. In this paper, we reviewed the most recent advances and challenges of cognitive radio technology especially in the wideband spectrum sensing research. We classified the sensing techniques into two main categories: narrowband and wideband spectrum sensing. Techniques of each category are then presented, and open directions discussed. This survey also discusses how cognitive radio technology can be incorporated in future networks to overcome the challenges related to access to the radio spectrum. Finally, we discuss some of the open research directions related to attacks targeting secure spectrum sensing and sharing.

## Figures and Tables

**Table 1:** Related work.

Related Work	Topic	Concepts Covered	Concepts Not Covered
S. K. Sharma [53]	Application of Compressive Sensing in Cognitive Radio Communications	Review of compressive sensing Analysis of the application of compressive sensing	Wideband compressive sensing techniques
F. Salahdine et al. [54]	Survey on compressive sensing techniques	Theory of compressive sensing Analytical Comparison of compressive sensing techniques Examples of compressive sensing of applications	Wideband spectrum sensing Wideband compressive sensing
Y. Arjoune et al. [56]	Survey of compressive sensing techniques	Analytical and quantitative comparison between compressive sensing techniques from several categories	Application of compressive sensing techniques
H. Sun et al. [61]	Survey of wideband spectrum sensing	Review of narrowband spectrum sensing techniques Review of wideband spectrum sensing High-level discussion of sub-Nyquist wideband sensing techniques	Compressive wideband sensing Blind compressive sensing Comparison of wideband sensing technique
T. Yucek et al. [68]	Survey on spectrum sensing techniques	Review of spectrum sensing methods Analytical comparison of narrowband sensing methods Cooperative spectrum sensing Spectrum sensing in current wireless standards	Wideband sensing Compressive sensing
L. De Vito [69]	Review of the spectrum sensing method	High-level discussion of wideband sensing techniques Brief discussion of cooperative sensing	Compressive sensing techniques Sparse basis selection Adaptive compressive sensing

**Table 2:** Machine learning for spectrum sensing in cognitive radio networks.

Related Work	Features	ML Algorithms	Evaluation Metrics
Madushan et al. [42]	Energy statistic	K-means Gaussian mixture model Support vector machine K-nearest-neighbor	Probability of detection Probability of false alarm Average training time
Zhang et al. [43]	Energy statistic	K-means Support vector machine	Discrimination probability Probability of detection Probability of false alarm
Khalfi et al. [44]	Occupancy over time	Linear Regression Support vector regression	Regression Time index Probability of false alarm Probability of false
Mikaeil et al. [45]	Energy statistic	K-nearest neighbor Support vector machine Naive Bayes Decision Tree	Probability of detection Probability of false alarm Accuracy Sensing time Delay
Lu et al. [46]	Probability vector	K-means cluster, SVM	Classification delay Probability of detection Probability of false alarm
Wang et al. [47]	Energy statistic	Random forest	Throughput Arrival rate
Ghazizadeh et al. [48]	Energy statistic	Supported vector machines K-nearest neighbors Naïve Bayes	Total error rate

**Table 3:** Advantages and disadvantages of the four narrowband spectrum sensing methods.

Sensing Technique	Advantages	Disadvantages
Energy detection [2,3,4,5,6,7,8,9,10,11,12,13,14,15,16,17,18,19,20]	Easy to implement No prior knowledge of the primary signal characteristics is required	High false alarm rate Unreliable at low SNR values Sensitive to noise uncertainty
Cyclo-stationary feature detection [21,22,23,24,25,26,27]	Robust against noise uncertainty Distinguish between signal and noise Decreased probability of false alarm at low SNR	Large sensing time to achieve a good performance High energy consumption when the size of the samples is large
Matched Filter based detection [28,29,30,31]	Better detection at low SNR region Optimal sensing	Prior knowledge of the primary user signal is required Impractical since prior knowledge about the signal is not always available
Covariance-based detection [32,33,34,35,36,37,38,39]	No prior knowledge of the primary user signal and noise is required Blindly detection	Good computational complexity coming
Machine learning based spectrum sensing [40,41,42,43,44,45,46,47,48,49,50,51]	Machine learning can detect if trained correctly can be a good approach Minimize the delay of the detection Use complex model in an easy manner	Complex techniques Has to be adapted in learning in very fast changing environments Features selection affects detection rate and adds complexity High dataset has to be build

**Table 4:** Advantages and disadvantages of wideband sensing techniques.

Wideband Sensing Technique		Advantages	Disadvantages
Nyquist-based techniques	Wavelet [71,72,73,74,75,76]	Reduced latency compared to single band detection	Unaffordable sampling rate High latency High energy consumption High complexity
	Multi-band joint detection [77,78]		
	Filter bank [79,80,81,82]	Reduced latency compared to wavelet-based sensing techniques	
Sub-Nyquist-based techniques	AIC [65]	Reduced number of measurements compared to Nyquist- based sensing techniques	Number of measurements to be used is not specified Reduced performance compared to non-compressed techniques
	Two-step CS [83,85]	Estimates the sparsity of the wideband signal, which allows the use of a suitable number of samples	More complexity due to the estimation bloc
	Geo-location CS [88]	Database stores the sparsity of the signal and the list of occupied frequency channels Sparsity estimation is not done to reduce the complexity	Database has to be updated in real time High latency due to the communication with the database
	Adaptive CS [87]	The number of samples is determined. The recovery error is controlled	Higher computational complexity since the recovery process has to be performed several times
	Compressive sensing with recovery [65,82,84,85,86,87,88,89]	Discrete Cosine Transform is more accurate than Discrete Fourier Transform Reduces processing time	Higher complexity and reduced performance compared to non-compressed techniques in terms of probabilities of detection and false alarm Estimation of sparsity level is needed
	Compressive measurements’ using DCT sensing matrix without recovery [71,72,73]	Reduced complexity Detection performed directly from the measurements, no recovery needed No sparsity estimation required	Reduced performance compared to compressive spectrum in terms of probabilities of detection and false alarm compared with Nyquist-based approach
	One-bit compressive sensing [99,100,101,102,103,104,105]	Fast sampling Low complexity Low computational cost Low storage cost and hardware complexity Robust to noise	Need to be investigated more in cognitive radio networks
